# Quantifying the handprint—Footprint balance into a single score: The example of pharmaceuticals

**DOI:** 10.1371/journal.pone.0229235

**Published:** 2020-02-18

**Authors:** Sam Debaveye, Delphine De Smedt, Bert Heirman, Shane Kavanagh, Jo Dewulf

**Affiliations:** 1 Department of Green Chemistry and Technology, Ghent University, Campus Coupure, Ghent, Belgium; 2 Department of Public Health, Ghent University, Campus UZ, Ghent, Belgium; 3 Johnson & Johnson Environment, Health, Safety & Sustainability, Janssen Pharmaceutica NV, Beerse, Belgium; 4 Health Economics, Janssen Pharmaceutica NV, Beerse, Belgium; Sciensano, BELGIUM

## Abstract

Life Cycle Assessment typically focuses on the footprint of products and services, expressed on three Areas of Protection (AoP): Human Health, Ecosystems and Resources. While the handprint is often expressed qualitatively, quantified handprints have recently been compared directly to the footprint concerning one AoP: Human Health. We propose to take this one step further by simultaneously comparing the quantified handprint and footprint on all AoPs through normalization and weighting of the results towards a single score. We discuss two example cases of a pharmaceutical treatment: mebendazole to treat soil-transmitted helminthiases and paliperidone palmitate to treat schizophrenia. Each time, treatment is compared to ‘no treatment’. The footprint of health care is compared to the handprint of improved patient health. The handprint and footprint were normalized separately. To include sensitivity in the normalization step we applied four sets of external normalization factors for both handprint (Global Burden of Disease) and footprint (ReCiPe and PROSUITE). At the weighting step we applied 26 sets of panel weighting factors from three sources. We propose the Relative Sustainability Benefit Rate (RSBR) as a new metric to quantify the relative difference in combined handprint and footprint single score between two alternatives. When only considering the footprint, the first case study is associated with an increased single score burden of treatment compared to ‘no treatment’, while in the second case study treatment reduces the single score burden by 41.1% compared to ‘no treatment’. Also including the handprint provided new insights for the first case study, now showing a decrease of 56.4% in single score burden for treatment compared to ‘no treatment’. For the second case study the reduction of single score burden was confirmed as the handprint burden was also decreased because of treatment by 9.9%, reinforcing the findings.

## Introduction

Sustainability is becoming an ever more important part of the political and research agenda, as illustrated by the United Nations Sustainable Development Goals launched in 2015 [1]. Sustainability covers many domains, and a multidisciplinary approach is required to holistically capture the three traditional pillars: People, Planet, Profit [2, 3]. When focusing on the Planet or the impact on the environment, Life Cycle Assessment (LCA) is one of the most mature methodologies to assess and quantify the environmental sustainability of products and services [4, 5]. LCA typically focuses on the (environmental) footprint, or the negative impact of companies, consumers and governments on the environment as a result of resource use and emissions. The counterpart of the footprint is the handprint, defined as the quantifiable impact of actions that have a positive impact on humans or the environment. From a footprint perspective, the aim is to strive for a reduction of the environmental impact towards zero. For the handprint however, the goal is either to prevent footprints that would otherwise have occurred, or create positive benefits that would otherwise not have occurred. The aim of the handprint is to increase the positive impact ever more, as there is no implied limit. By including the handprint next to the footprint, products and services are not only evaluated on their environmental impact but also on the positive actions associated with the functional unit, i.e. the unit of comparison. When the negative impacts of the footprint are overcompensated by the positive impacts from the handprint, the aim of a ‘net positive’ result is achieved. While LCA provides a comprehensive quantification of the environmental footprint on the three main impact categories or Areas of Protection (AoP) Human Health, Ecosystem Quality and Natural Resources, the benefit or handprint of products and services is typically only qualitatively represented by the functional unit [6–12].

This changed in recent years as LCA practitioners are starting to include a quantified handprint to the otherwise footprint-oriented approach [13–18]. When focusing on one Area of Protection, it is possible to directly compare the handprint to the footprint [15, 16, 18–20]. For example, Arvidsson et al. (2016) compare the Human Health burden associated with the production of airbag systems with the avoided casualties and injuries from car crashes.

However, this excludes the other two AoPs. We propose to take this one step further, by simultaneously comparing the quantified handprint on Human Health to the footprint on all three AoPs.

To do this, multiple aggregation approaches exist, such as monetization, Distance-to-Target, Multi-Criteria Decision Analysis (MCDA) and normalization and weighting [21–30]. For normalization, a distinction can be made between internal or external normalization [31]. These approaches enable the aggregation of the results towards a single score metric. This allows a more holistic sustainability comparison of two alternatives, both from an absolute and relative perspective.

In this study we discuss two example cases of pharmaceutical treatment from the health care sector: an anthelmintic for the treatment of children with a soil-transmitted helminthiases (STH) worm infection in Vietnam and an antipsychotic for the treatment of patients with schizophrenia in Belgium [32, 33]. The handprint of the pharmaceutical treatment of patients can be quantified through multiple metrics such as the Quality-Adjusted Life Year (QALY) or Disability-Adjusted Life Year (DALY) [34, 35]. The latter can be directly compared to the AoP Human Health expressed in DALY, as this was developed to be the same metric [36–38].

The aim of this study is to holistically quantify and compare the handprint on Human Health and the footprint on three Areas of Protection of two pharmaceutical treatments to ‘no treatment’, by aggregating these into a single score using normalization and weighting. This approach shows what the impact of including the handprint can be on the single score burden, as opposed to only considering the footprint. It also provides an answer to the question: does the handprint outweigh the footprint? The approach can be adopted to assess any other product or service with a quantifiable handprint and footprint on one or more AoPs.

## Material and methods

### 2.1 Overall framework

**Fig 1:**
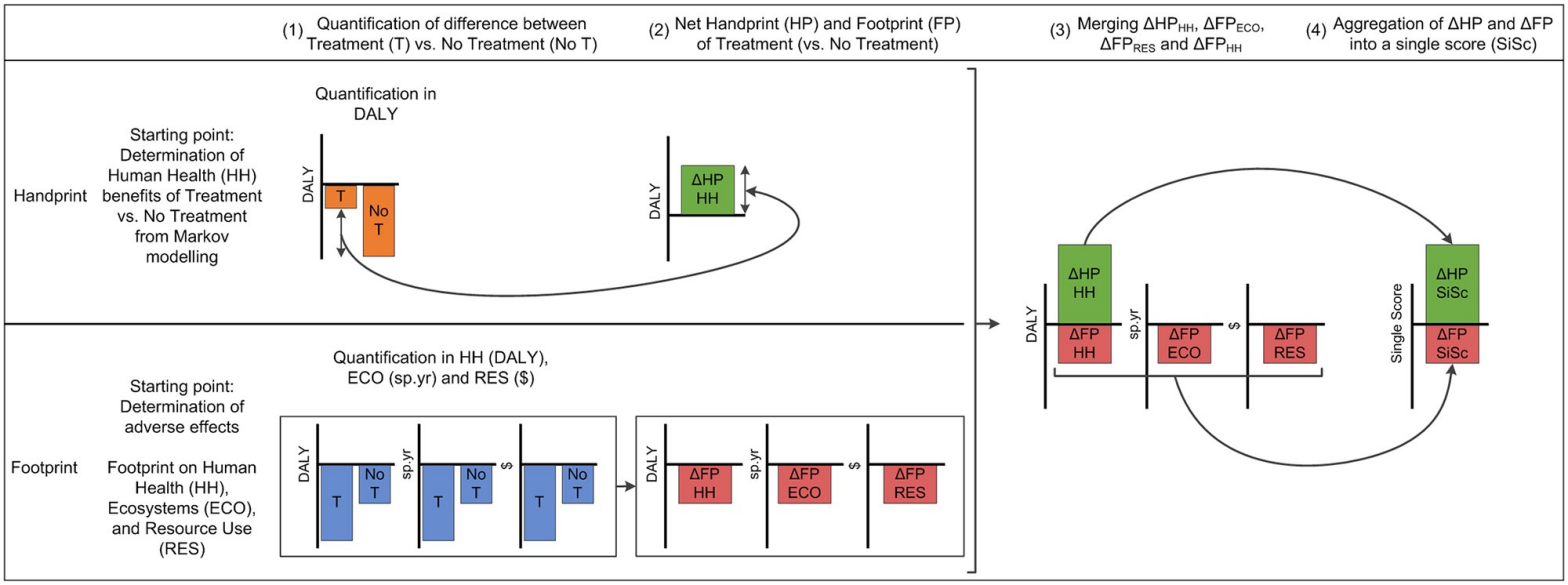
The overall framework applied in this study. The functional unit of a pharmaceutical treatment is used to show how the handprint could be added to the footprint. Abbreviations: T, Treatment; No T, No Treatment; HH, Human Health; ECO, Ecosystems; RES, Resources; SiSc, Single Score; DALY, Disability-Adjusted Life Year; sp.yr, species.year.

The overall framework consists of three main parts, represented in Fig 1. First, on the lower left side of Fig 1, we quantify the full cradle-to-grave environmental footprint of the two pharmaceutical treatments on the three AoPs, which is compared to the footprint of a ‘no treatment’ scenario [39]. The values presented in the figure are examples to illustrate the concept. The delta (Δ) value is the difference between treatment and ‘no treatment’. Note that ‘no treatment’ means no *pharmaceutical* treatment, and can still represent an environmental impact through e.g. hospital stays.

Second, on the upper left side of Fig 1, we include a quantitative handprint, as opposed to the traditional qualitative functional unit [8]. This handprint is expressed in the same metric as one of the AoP, in this case also Human Health. This enables a direct comparison between both the Human Health (HH) handprint_HH_ and footprint_HH_ [10, 40–42]. Third, on the right side of Fig 1, we aggregate the handprint and footprint on all AoPs towards a single score (SiSc) by applying multiple sets of external normalization and weighting factors, capturing various perspectives with regard to value choices [43–46]. This enables a direct comparison of the handprint_SiSc_ and footprint_SiSc_ on all three AoPs for both treatment and ‘no treatment’ scenarios.

The Δhandprint is visualized in Fig 1 as a positive value, which is often the case when comparing treatment to ‘no treatment’. Both QALY’s and DALY’s could have been used to quantify the handprint in this study. The DALY was chosen as it is directly comparable to the DALY used in LCA to quantify Human Health damage associated with resource use and emissions. Indeed, the DALY was introduced in LCA in by Hofstetter, based on earlier work by Murray and Lopez to quantify the Global Burden of Disease (GBD) and so is meant to be the same metric [39, 47, 48]. However, for treatment or ‘no treatment’ separately the patient health is expressed in DALYs, which is inherently a negative metric as it represents morbidity and mortality [48]. The positive value (DALYs avoided) is the result of the mathematical difference in handprint_HH_ between treatment and ‘no treatment’. Throughout this study, both the footprint_HH_ and handprint_HH_ for treatment and ‘no treatment’ will be expressed in terms of burden. The treatment benefit arises when comparing the burden of treatment against ‘no treatment’.

The single score, expressed in points, enables the calculation of both the absolute and relative difference in combined handprint_SiSc_ and footprint_SiSc_. For the latter we propose the Relative Sustainability Benefit Rate (RSBR) as a new metric to compare the relative difference in sustainability between two alternatives, based on a single score sustainability result. The RSBR is calculated using the following equation:

$$RSBR=\frac{{{(handprint}_{SiSc}+{footprint}_{SiSc})}_{no\;treatment}-{{(handprint}_{SiSc}+{footprint}_{SiSc})}_{treatment}}{{{(handprint}_{SiSc}+{footprint}_{SiSc})}_{no\;treatment}}*100(\%)$$

If the treatment has a single score of 90 and ‘no treatment’ scores 100, then treatment is 10 point better or has a RSBR of 10%. The RSBR can also be negative, if the treatment performs worse than ‘no treatment’. The value of the RSBR lies in its interpretation: when results cannot be presented as easy integers of 90 and 100 then a comparison in absolute numbers can rapidly become complex. Therefore a simple outcome in percentage can help to communicate the results of the study.

### 2.2 Goal & scope

The goal of this study is to quantify the handprint on Human Health and the footprint on three AoPs for two pharmaceutical treatments compared to ‘no treatment’. Both are then aggregated towards a single score by normalizing to make the results dimensionless and weighting as a value judgement. First we calculate the results when only considering the footprint, after which the handprint is added to show the difference between both approaches. After normalization and weighting of the results towards a single score, we calculate the absolute and relative difference (RSBR) in combined handprint_SiSc_ and footprint_SiSc_. The scope of the two pharmaceutical treatments will be discussed in the next sections.

The functional unit of the first case study is the treatment of soil-transmitted helminthiases (STH) worm infection in 8 million Vietnamese children aged 5–14 with mebendazole (500 mg) every six months for five years, reaching 80% coverage. This is the Mass Drug Administration program as recommended by the WHO and implemented in Vietnam [49, 50]. This scenario is compared to ‘no treatment’.

The environmental footprint is based on the resource use and emissions directly associated with the pharmaceutical supply chain of mebendazole. This includes the Active Pharmaceutical Ingredient (API) synthesis, tablet formulation, packaging, distribution to Vietnam and End-of-Life of the molecule and packaging materials. For ‘no treatment’ there is no pharmaceutical supply chain to consider. We assumed that no health care providers such as hospitals (visits) are directly associated with STH. Therefore no environmental impact was included for ‘no treatment’.

The handprint considers anthelmintic treatment which reduces the intensity of worm infection and leads to a reduction in Human Health disability due to STH infection over time. A published Markov model developed to monitor MDA programs for school-aged children was adopted to simulate the estimated reduction in STH prevalence due to mebendazole MDA [51, 52]. A Markov model is a state-transition model with multiple (health) states. Over multiple cycles, a modelled individual can move from one state to another with a certain defined probability, which makes it useful to model diseases where there is a probability of recurrence or repeated events. The average number of cycles that individuals reside in each state can be linked to a health value per state [53]. The model we adopted was Transition Probability Matrix Set (TPMS) 4 from the Supplementary Material of Montresor et al. (2016), representing the treatment of 1324 school-aged children with mebendazole every 12 months. As we aimed to model the effect of treatment with mebendazole every six months, as recommended by the WHO when STH prevalence is higher than 50% and which was actually (partly) implemented in Vietnam, the results of TPMS 4 are considered conservative [54, 55]. The model was linked to disability weights of the Global Burden of Disease (GBD) [51, 52, 56–61]. The treatment is based on a model that quantifies the reduction in disability over five years, while ‘no treatment’ assumes that the initial worm infection stays constant over time.

The functional unit of the second case study concerns the treatment of 1000 patients with schizophrenia for one year with the long-acting (30 days) injectable antipsychotic paliperidone palmitate (100 mg) in Belgium [32]. This is compared to Treatment Interruption (TI), as ‘no treatment’ of patients with schizophrenia in Belgium has not been part of the standard of care for decades.

The environmental footprint is based on the resource use and emissions directly associated with the health care pathway, including the pharmaceutical supply chain of the medicine and the related health care providers. The former includes API synthesis, drug production, packaging, distribution and End-of-Life of the molecule and packaging materials. The latter includes the associated general and psychiatric hospitals stays, General Practitioner and psychiatrist visits and ambulatory care.

The handprint considers the effect of antipsychotic treatment which keeps patients more stable, reducing the probability of an acute psychotic episode or relapse. This leads to a reduction in Human Health disability of the treated patients and is based on a Markov model that simulates treatment over one year [32, 58, 62–70]. A new Markov model was developed to simulate disease outcomes of patients for PP1M and PP3M treatment, as well as Treatment Interruption. The population is a hypothetical Belgian patient cohort eligible for the maintenance treatment of schizophrenia [71–73]. Transition probabilities for the model were based on nine literature reviews. Real-world evidence was used when available and secondary analysis was performed, for instance to isolate the patient group diagnosed with schizophrenia.

For a more detailed interpretation of the handprint and footprint of both pharmaceutical treatments, more information on the study scope, inputs, methods and results can be found in Debaveye et al. (2018) and Debaveye et al. (2019) [74–75].

Due to the difference in functional unit between both case studies regarding the number of patients (8 million vs. 1000) and time horizon (5 vs. 1 years), the handprint and footprint data will be of a different order of magnitude, something the reader should keep in mind when interpreting the results.

Note that there is also a marked difference in scope between handprint and footprint from a spatial, time and person perspective. In both cases individual patients are treated in one country with a time horizon of a few years. The environmental impact manifests on a regional or even global scale and may continue for decades or centuries [19, 43, 44]. This extended time horizon of environmental stressors is internalized in LCA modelling [39].

### 2.3 Life cycle inventory

The Life Cycle Inventory (LCI) of the two pharmaceutical treatments was performed for the resource use and emissions of the pharmaceutical supply chain and the health care providers. Primary data, i.e. data gathered on the foreground processes by the authors, was used for all parts of the LCI. Secondary data for the background processes (the processes that support the foreground) such as energy and chemicals were extracted from the ecoinvent v3.1 database using SimaPro v8 software [76, 77].

Primary data on the pharmaceutical supply chain was gathered from Janssen Pharmaceutica, and their external supply partners. The system boundaries were the limits of the production plants, however transport of intermediate products between plants was included, as well as the final transport to the patients. We included the resource use of industrial unit operations as well as the plant supporting processes. The electricity mix was adapted concerning the origin of the electricity generation per production site. The LCI of the health care providers, consists of the average transport distance and the on-site energy and water use of hospitals. Further details can be found in Debaveye et al. (2018) and Debaveye et al. (2019).

We separately calculated the uncertainty on the Life Cycle Inventory data present in the ecoinvent database by running an uncertainty analysis in SimaPro v8 for 1000 iterations [77–79]. This uncertainty was not included in the calculation of the RSBR, as we wanted to focus on the influence of using different normalization and weighting sets.

### 2.4 Life cycle impact assessment

#### 2.4.1 Part 1: Quantification of the footprint

The first part of the impact assessment concerns the environmental footprint on three AoPs of the pharmaceutical treatments. For this, we used the ReCiPe Endpoint Hierarchist v1.11 method, which is an impact assessment method that translates resource use and emission to environmental impacts. The method was implemented through SimaPro v8 software, which facilitates LCA by allowing a combination of different LCA resource and emission databases and life cycle impact methods. The impact assessment of the End-of-Life fate of the pharmaceutical molecules considered emissions to continental freshwater using the USEtox methodology [80, 81]. The aggregated results of the impact assessment can be found in Table 1 while the full results can be found in Supporting Information, S1 and S2 Tables in S1 File.

**Table 1:** Footprint of the pharmaceutical treatments on three Areas of Protection.

	Human Health	Ecosystems	Resources
	DALY	species.yr	$
**Disease area: soil-transmitted helminthiases (Vietnam)**			
(FU: 8,000,000 children, 5 years)			
‘No treatment’	NA	NA	NA
Treatment: mebendazole 500 mg every six months	5.75E+00	2.87E-02	2.23E+05
**Disease area: schizophrenia (Belgium)**			
(FU: 1000 patients, 1 year)			
Treatment Interruption	8.55E-01	5.00E-03	3.35E+04
Treatment: paliperidone palmitate 100 mg once-monthly	5.12E-01	2.92E-03	1.93E+04

Abbreviations: FU, functional unit; DALY, Disability-Adjusted Life Year; NA, not assessed; yr, year

At the endpoint level, damage is modelled to areas that society wishes to safeguard: Human Health, Ecosystems and Resources. Damage to Human Health is expressed as Disability-Adjusted Life Years (DALY), a metric to quantify disease burden developed by the World Health Organization (WHO) that combines disability with life expectancy [48]. Damage to Ecosystems diversity is expressed in species.yr, or the potential number of species that disappear each year as a result of disruption by anthropogenic activities [82]. Specifically for mebendazole MDA, the treatment-related reduction in STH prevalence is excluded and, if it were included, would not count as extra damage as none of the STH worm species are extinct. Resources are expressed in US dollar ($), representing the surplus cost or marginal increase of the extraction cost for society of the next batch of resources, which increases as the more readily available resource deposits are depleted [83].

For the donation of mebendazole the bulk of the environmental impact is associated with the synthesis of the Active Pharmaceutical Ingredient (API) while tablet formulation is the second largest contributor.

For the treatment of schizophrenia with paliperidone palmitate, the main environmental impact was associated with psychiatrist visits and general and psychiatric hospital stays. Because treatment keeps patients with schizophrenia more stable, they perform less days in the hospital. From an environmental point of view, the added impact of treatment associated with the pharmaceutical supply chain is overcompensated by a decrease in hospital stays. Hence, the environmental impact of treatment is lower than that of Treatment Interruption (Table 1).

#### 2.4.2 Part 2: Quantification of the handprint

The second part considers the quantified handprint on the AoP Human Health. For both pharmaceutical treatments the Human Health burden in DALY is compared for the treatment and ‘no treatment’ scenarios [48]. As can be seen in Table 2, treatment improves patient health, which is reflected in lower DALYs for the treatment groups. The minimum and maximum values were obtained from a sensitivity analysis of the specific treatment models, and are included in the sensitivity analysis in this study.

**Table 2:** Human health handprint of the pharmaceutical treatments.

	DALY base case	DALY min.	DALY max.
**Disease area: soil-transmitted helminthiases (Vietnam)**			
(FU: 8,000,000 children, 5 years)			
‘No treatment’	206,533	98,819	336,790
Treatment: mebendazole 500 mg every six months	89,946 (-67.7%)	58,812	124,840
**Disease area: schizophrenia (Belgium)**			
(FU: 1000 patients, 1 year)			
Treatment Interruption	973.03	942.08	1,017.71
Treatment: paliperidone palmitate 100 mg once-monthly	904.12 (-7.1%)	889.54	920.16

Abbreviations: FU, functional unit; DALY, Disability-Adjusted Life Year

Before treatment of soil-transmitted helminthiases with mebendazole, the 8 million children have a relatively high intensity of worm infection. Disability due to STH is highly dependent on the intensity of infection which is reduced by treatment. Therefore treated children have a 67.7% benefit in handprint_HH_ than if they would not have been treated, represented by ‘no treatment’ [51, 57].

For schizophrenia, patients frequently experience an acute psychotic episode or relapse, where they have to recover from in the hospital or through intensive ambulatory care. The probability for a relapse is reduced by treatment, keeping patients more stable, enabling them to live fuller lives and leading to a 7.1% benefit in handprint_HH_ compared to Treatment Interruption [32, 69]. Note the magnitude difference of the DALYs, which is due to the number of patients under study: 8 million vs. 1000.

#### 2.4.3 Part 3: Normalization and weighting towards a single score

The third part is the normalization and weighting of the footprint and handprint. Normalization allows the direct comparison of results across multiple impact assessment categories (that have different units) by adjusting the results towards common dimensions [31]. There are two main normalization methods: external and internal normalization. External normalization divides the impacts in the study by the total amount of that impact category for a given region and year, e.g. the total DALYs as a result of environmental impact in Europe in the year 2000. That way, external normalization allows the quantification of the relative significance of impact categories. Internal normalization on the other hand compares only the impacts under study, and for example ranks the three alternatives between 0 and 1. That way, internal normalization does not quantify the relative significance of the impact from a wider perspective.

Both handprint and footprint were normalized separately and for each a set of multiple external Normalization Factors (NF) was used, as we observe there is no normalization approach that allows simultaneous assessment of both handprint and footprint. Although both handprint_HH_ and footprint_HH_ have the same unit, they are normalized separately because the handprint considers a direct Human Health burden to patients, while the footprint is associated with the yearly fraction of Human Health burden associated with environmental factors [84]. Indeed, both the handprint and the footprint were normalised by the total global impact of their respective cause-effect chain. In the footprint cause-effect chain, the different types of resource use and emissions are aggregated to a certain smaller set of reference compounds, e.g. CO_2_-equivalents. The impact of the reference compounds on environmental impact categories, such as Ecosystems and Human Health, is then calculated [85–87]. For the handprint, the cause-effect chain is considered to start from each disease, multiplying the prevalence with the health loss in DALYs caused by the disease per afflicted person [58, 88].

Specifically for the Area of Protection Human Health, the footprint is the result (cause-effect-wise) in health damage attributable to environmental factors, therefore it is normalised with the total health damage attributable to the environment. The handprint however has a completely different cause-effect chain, if even quantifiable (for all diseases), and therefore it was normalized with the total global health damage from all diseases. Furthermore, one of the goals of normalization is to quantify and compare the relative significance of multiple results.

The footprint was normalized with the three global NF sets of the year 2000 in the ReCiPe Endpoint 1.11 method: Individualist, Hierarchist and Egalitarian and with the global NF set developed in the PROSUITE project (European Commission 7^th^ Framework Programme project which aimed for the development and application of a standardized methodology for the prospective sustainability assessment of technologies) for the year 2010 (Supporting Information, S3 Table in S1 File) [89, 90]. For Human Health, only the burden induced by environmental factors is reflected in the NF [39, 84]. The handprint was normalized with the global Human Health burden reported by the GBD at 5-year intervals from 2000 to 2015 [41] (Supporting Information, S4 Table in S1 File). Global NF were chosen as pharmaceutical treatments in multiple continents are considered [91].

To include the sensitivity on the choice of NF, the results of both case studies were calculated 10,000 times. Each iteration, a random NF for the footprint was chosen from S3 Table in S1 File, Supporting Information. At the same time, a random NF for the handprint was chosen from S4 Table in S1 File, Supporting Information.

To aggregate all AoPs to a single score, weighting was applied through three main sets of panel Weighting Factors (WF). Multiple weighting methods exist [24, 28]. The panel weighting method develops weighting sets based on the value choices of a panel of experts or laymen. Each respondent in the panel is asked by means of questionnaires or group discussions how important they consider all impact categories compared to each other [29, 92]. The sum of all weights is always 1. The first main set was adopted from Eco-Indicator 99, including four weighting sub-sets: default, Individualist, Hierarchist and Egalitarian [29]. The second main set was selected from the work of Itsubo et al. (2015), where we included weighting sub-sets from 19 countries, representing all continents [92]. The third was adopted from the technical report of the European Commission (EC) Joint Research Centre (JRC) on weighting factors for the Environmental Footprint (EF) [93].

A full list of weighting factors can be found in Supporting Information, S1-S3 Figs, S5 Table in S1 File. Human Health received the highest weight in 16 out of 26 WF sets. Ecosystems was the highest in 8 WF sets, while an equal weight was given to Human Health and Ecosystems in 2 WF sets. Resources was never given the highest weight. This study focuses in the first place on testing the robustness of the results with multiple readily available WF, rather than identifying the ‘best’ WF set. When multiplying the four NF for the handprint_HH_, four NF for the footprint, 26 WF and the three options for the handprint on patient DALYs (base case, min, max) there are 1248 possible combinations.

For each of the 10,000 iterations that started with normalization, a choice was then made for one of the three main sets of WF, which are represented by the three columns in S5 Table in S1 File, Supporting Information. Each of the columns or main sets of WF had an equal probability of providing the WF for that iteration. Out of that main WF set, a sub-set was then randomly selected, each with equal probability. For example, there is a 33,33% probability that Eco-indicator 99 is chosen as the main WF set for a certain iteration. Within that main WF set, there is a 25% probability that the Hierarchist sub-set is chosen as the final WF. A separate One-Way Sensitivity analysis of 2,000 iterations was performed on the effect of either normalization or weighting on the single score results.

Only after normalization and weighting were the handprint and footprint combined for the AoP Human Health. After that, the dimensionless scores for the three AoPs were aggregated to a single score burden. For each separate iteration, the absolute and relative difference between the single score results was calculated. For the latter, we used the RSBR formula as discussed earlier in section 2.1.

#### 2.4.4 Applying the RSBR concept to both cases

As can be seen from Tables 1 and 2, there is a crucial difference between the two case studies when considering the footprint of treatment vs. ‘no treatment’ or vs. Treatment Interruption. For the case study on STH, the treatment leads to an increase in footprint, which can be overcompensated by the decrease in burden on the handprint. This way, the RSBR expresses the full benefit of treatment, weighting a better handprint against an increase in footprint. For the case study on schizophrenia however, there is a decrease in burden both for the footprint and the handprint. Hence, the RSBR will be positive for both handprint and footprint separately, and a weighted sum of both towards an aggregate RSBR may not be appropriate. When comparing treatment to Treatment Interruption, the handprint has a larger *absolute benefit* than the footprint, while the *relative benefit* of the handprint is smaller than that of the footprint. As the handprint dominates the footprint in absolute magnitude, even after normalization, this would mean that the RSBR would reflect more the smaller relative benefit of the handprint, which does not do justice to the larger relative avoided burdens of the footprint. Therefore, the RSBR should be kept separate for the handprint and the footprint if both are increasing or decreasing, when comparing treatment vs. ‘no treatment’.

## Results

**Fig 2:**
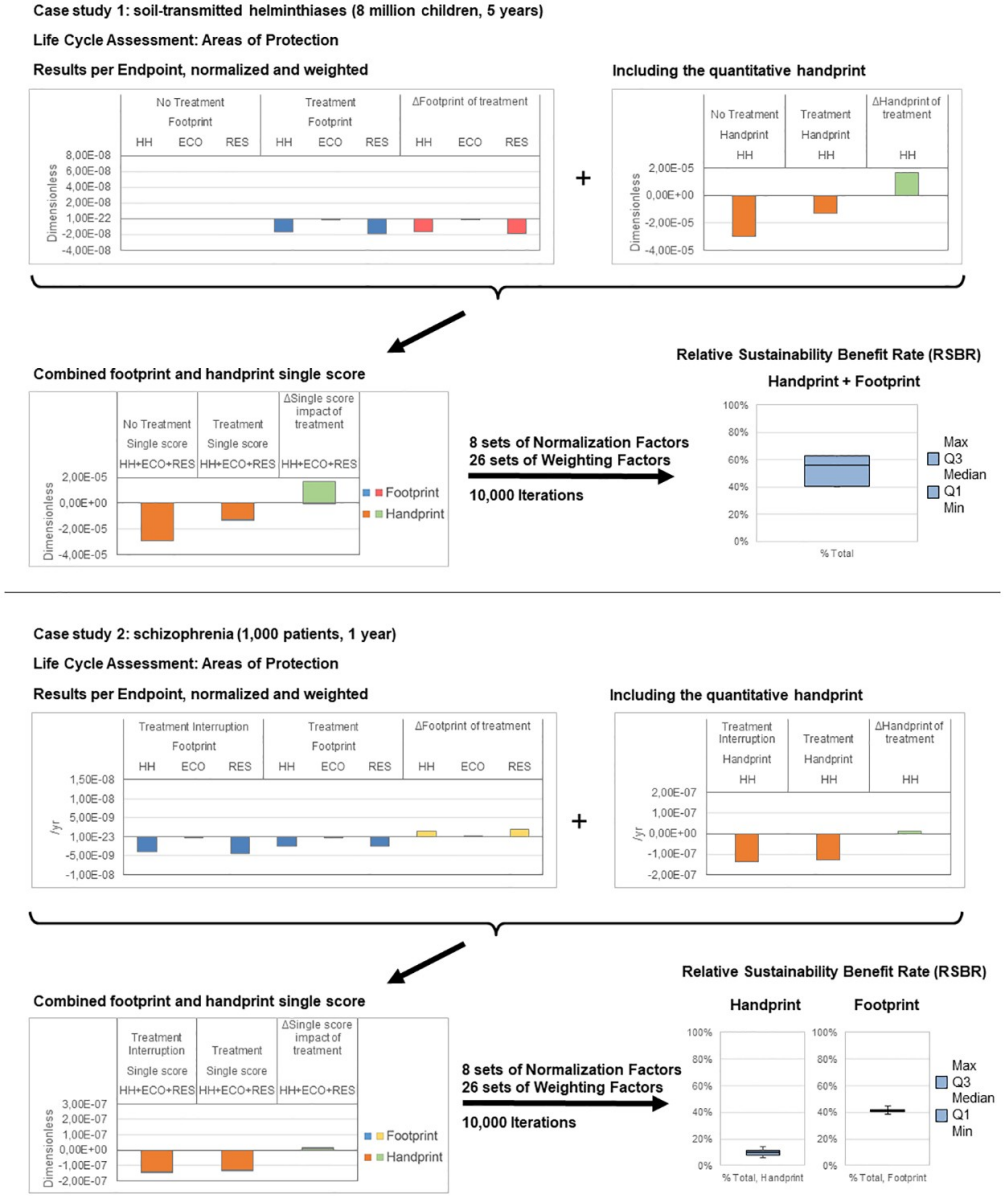
Normalization and weighting of footprint and handprint towards a single score for the first (a) and second (b) case studies on respectively soil-transmitted helminthiases and schizophrenia. Abbreviations: HH, Human Health; ECO, Ecosystems; RES, Resources.

The results are presented in Table 3 and Fig 2. To make the table and figures more accessible, the results of only one set of normalization and weighting factors are presented. The NF sets for the footprint and handprint are respectively ReCiPe Hierarchist World 2000 and GBD World 2000, the WF set is the Eco-Indicator 99 default. All combinations of NF and WF were applied and resulted in the sensitivity on the RSBR value.

**Table 3:** Normalization and weighting of the footprint and handprint towards a single score.

	After normalization			After weighting			Single score	
	(ReCiPe Hierarchist World 2000, GBD World 2000)			(Eco-Indicator 99 Default)				
	Human Health	Ecosystems	Resources	Human Health	Ecosystems	Resources		
	/yr	/yr	/yr	-	-	-	-	RSBR
**Disease area: soil-transmitted helminthiases (Vietnam)**								
(FU: 8,000,000 children, 5 years)								
**Footprint**								
‘No treatment’	0.00E+00	0.00E+00	0.00E+00	0.00E+00	0.00E+00	0.00E+00	0.00E+00	
Treatment: mebendazole 500 mg every six months	6.94E-08	5.15E-09	1.50E-07	2.77E-08	2.06E-09	2.99E-08	5.98E-08	NA
**Handprint + footprint**								
‘No treatment’	7.38E-05	0.00E+00	0.00E+00	2.95E-05	0.00E+00	0.00E+00	2.95E-05	
Treatment: mebendazole 500 mg every six months	3.22E-05	5.15E-09	1.50E-07	1.29E-05	2.06E-09	2.99E-08	1.29E-05	-56.25%
**Disease area: schizophrenia (Belgium)**								
(FU: 1000 patients, 1 year)								
**Footprint**								
Treatment Interruption	9.81E-09	8.97E-10	2.25E-08	3.93E-09	3.59E-10	4.50E-09	8.79E-09	
Treatment: paliperidone palmitate 100 mg once-monthly	6.18E-09	5.24E-10	1.30E-08	2.47E-09	2.09E-10	2.60E-09	5.28E-09	-39.9%
**Handprint**								
Treatment Interruption	3.48E-07	NA	NA	1.39E-07	NA	NA	1.39E-07	
Treatment: paliperidone palmitate 100 mg once-monthly	3.16E-07	NA	NA	1.27E-07	NA	NA	1.27E-07	-9.02%

Abbreviations: NA, not-applicable

Table 3 shows the results after normalization (/yr), after weighting (dimensionless) and as a single score (dimensionless) for the two case studies. The first part of the results only considers the footprint. The second part adds the handprint to the results, combining handprint and footprint in the first case study, while in the second case study the footprint and handprint are kept separate.

The upper parts of both sections of Fig 2 show the separate results after normalization and weighting of the footprint and handprint. On the lower part of the sections, the handprint and footprint are summed towards a combined single score result for the first case study, with the sensitivity on the RSBR in the lower right part of the sections.

When only considering the footprint for the first case study on STH, the treatment has an environmental impact on all AoPs compared to the assumed burden-free reference ‘no treatment’ scenario, represented by the transition from blue to red in the figures. For the second case study on schizophrenia, the treatment single score burden is 39.9% lower than that of Treatment Interruption, represented by the transition from blue to yellow in the figures. Treatment keeps patients with schizophrenia more stable, reducing the probability of an acute psychotic episode or relapse. As patients recover from relapses with the aid of health care providers such as hospitals, treatment reduces the environmental impact associated with these services. From an environmental point of view, the added impact of treatment associated with the pharmaceutical supply chain is overcompensated by a decrease in hospital stays.

For the AoP Human Health the midpoint indicator Climate Change is responsible for the largest share of environmental impact: respectively 69.1% and 80.9% for the first and second case studies. Climate Change is also the largest contributor for the AoP Ecosystems: respectively 87.3% and 79.4% for the first and second case studies. The AoP Resources is dominated by the environmental impact from midpoint indicator Fossil Depletion: respectively 99.8% and 99.5 for the first and second case studies.

The uncertainty analysis on the Life Cycle Inventory based on the ecoinvent database showed that the impact assessment results for the three AoPs vary less than 25% for the first case study and less than 50% for the second case study, considering the 2.5–97.5 percentiles. Only for the second case study did the AoP Resources have a slightly higher uncertainty, varying 61% up and 35% down.

Including the handprint causes a considerable 56.4% (median) decrease, with min. and max. values of respectively 40.3% and 62.9%, in the total single score burden for the first case study, represented by the transition from orange to green in the figures. It was mentioned earlier for the second case study that, compared to only including the footprint, the handprint drives the results towards a larger absolute difference of treatment vs. Treatment Interruption. However, the relative difference is reduced. Therefore we report the RSBR ratios for the handprint and footprint separately. The handprint has a median RSBR of 9.9%, with a min. and max. value of 6.0% and 14.3%. The footprint has a median RSBR of 41.1%, with min. and max. values of 38.6% and 41.9%. Again note the difference in magnitude on the dimensionless axes between both case studies, which is due to the number of patients under study: 8 million vs. 1000.

When focusing on the part of the single score associated with Human Health for the first case study, 99.8% for the treatment and 100% for ‘no treatment’ is associated with the handprint. For the second case study this is 98.1% for the treatment and 97.3% for Treatment Interruption. This shows that the handprint is dominant to the footprint in absolute single score numbers.

**Fig 3:**
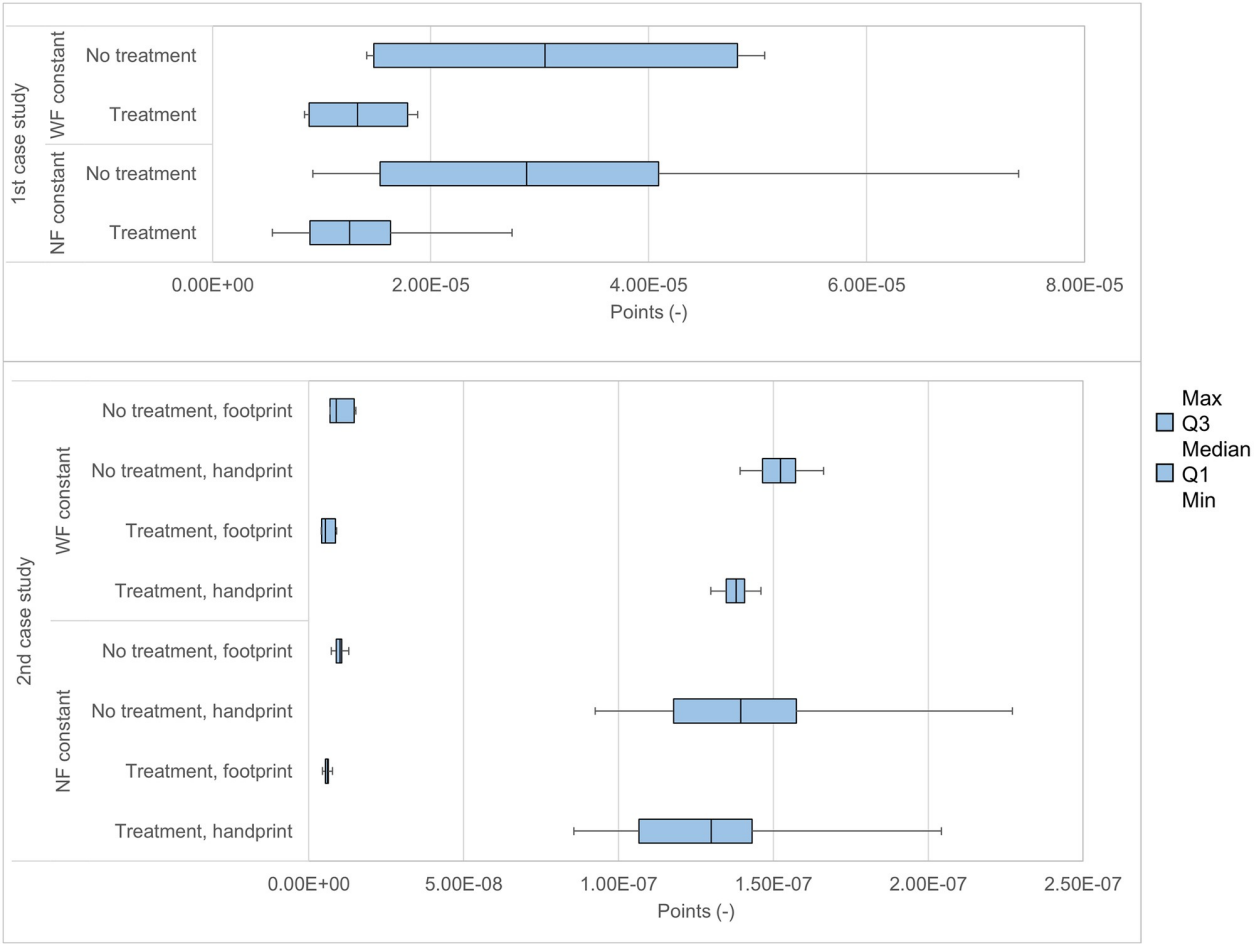
One-Way Sensitivity analysis of the single score results by keeping constant either the Normalization Factors (NF) or the Weighting Factors (WF), expressed in dimensionless points.

We considered the influence of either normalization or weighting on the absolute single score results through a One-Way Sensitivity analysis, of which the results are displayed in Fig 3. For both case studies, the single score results vary more when keeping the Normalization Factors constant, than when keeping the Weighting Factors constant. Thus, the Weighting Factors are responsible for more variability in the single score results than the Normalization Factors.

There is a large difference in RSBR results between treatment and ‘no treatment’ of the two case studies. As this concerns two different disease areas from different countries and health care settings, this was not considered surprising.

## Discussion

### 4.1 Statement of principal findings

When only considering the footprint, the first case study is associated with an increased single score burden of treatment compared to ‘no treatment’, while in the second case study treatment reduces the single score burden by 41.1% compared to Treatment Interruption. By adding a quantified handprint to the footprint-oriented approach of classical LCA and directly comparing both after aggregating towards a single score, the results of the first case study changed significantly. For the case study on STH the extra information of the handprint provided new insights, now showing a decrease of 56.4% as opposed to an increase in single score results for the treatment compared to ‘no treatment’. In the second case study on schizophrenia, the treatment already showed a decrease in single score results compared to ‘no treatment’ in the footprint assessment. As discussed earlier, we kept the assessment of the handprint separate from the footprint. For the handprint, a median RSBR of 9.9% was reported, reinforcing the findings. The single score results are dominated by the Human Health burden of the handprint, while environmental burdens have limited impact.

### 4.2 Strengths and weaknesses of the study

To the best of our knowledge, this is the first attempt to directly compare the Human Health handprint of a pharmaceutical treatment to the footprint on all three AoPs through normalization and weighting of the impacts towards a single score. Two example cases of pharmaceutical treatments were adopted in this study, both including a full cradle-to-grave footprint on all three AoPs. The handprint of the treatment was directly comparable to the footprint on the AoP Human Health. The results of the two cases illustrate the potential of including a quantified handprint in future assessments.

The RSBR represents a simple outcome: how much better (%) is treatment to ‘no treatment’, simultaneously considering both the handprint and footprint. It is also possible to subdivide this per AoP: how much better is one alternative to another for Human Health, Ecosystems or Resources? This approach covers the environmental and social pillars of the sustainability triangle to some extent, while economic factors were outside the scope of this study [3]. A limitation of the RSBR concept is that it is most appropriate when comparing a decrease in burden for the handprint (of treatment vs. no treatment) to an increase in burden for the footprint. When this is not the case, RSBR values can be reported separately for the handprint and the footprint.

The handprint in this study was based on observed patient outcomes from standardized studies. This contrasts with the footprint, which is calculated from global models based on assumptions. Future assessments should appreciate this difference in data robustness.

We applied external normalization in this study for the purpose of directly comparing the relative significance of the handprint and footprint, which is not possible with internal normalization [31]. The NF for the handprint were at least 11.5 times larger than those of the footprint. As the handprint still dominates the results, even after normalization, the use of different (larger) NF for the handprint, compared to the footprint, was conservative.

Although the external normalization factors were the driving factor of the absolute results of the handprint vs. footprint comparison, the One-Way Sensitivity analysis showed that the weighting factors were responsible for more variability in the single score results than the normalization factors. The uncertainty inherent to the Life Cycle Inventory was considered separately from the main analysis. Given the limited uncertainty on the AoPs, this would have had a negligible impact on the RSBR results.

It should be tested if the findings hold when applying a different methodology, e.g. Multi-Criteria Decision Analysis (MCDA) or internal normalization [27, 31, 91]. Global Normalization Factors for the ILCD are currently tentative and were not included in this study [46].

One type of weighting was applied in this study: panel weighting. This is a compensatory approach, part of the ‘weak’ sustainability concept, which considers weights as trade-offs between the different Areas of Protection [94]. This means that a loss in one AoP can be compensated with a gain in another AoP, rather than only quantifying the relative importance of each AoP. Different weighting sets can thus be seen as multiple compensatory sets. While the authors chose for the transparency and inherent value judgement of the latter method, Distance-to-target weighting and monetization could also be applied [21, 24–26, 28, 30, 95–97].

In this study, a positive handprint_HH_ seems to be subjected to LCA-based weighting sets that actually consider the AoP Human Health as a footprint or burden. However, the weighting of the handprint occurs before the comparison of treatment vs. ‘no treatment’. At that moment, both handprints are still considered as burdens. The positive value only emerges after weighting, when the burden of ‘no treatment’ is compared to a reduced burden for treatment, leading to the positive value.

Dissemination of sustainability assessments typically suffers from the multitude of indicators involved, frequently showing an increase in one area while also noting a decrease in another. The alternative single score approach proposed in this study avoids a trade-off between two indicators with different units and provides a clear outcome. However, we acknowledge that this single score approach may not be appropriate in all cases and should therefore not replace sustainability assessments that take into account multiple indicators.

The use of normalization and weighting is a subject of debate in Life Cycle Assessment. For normalization the main issue is choosing between internal or external normalization [31]. Specifically for weighting, the policy has changed over the years with older International Organization for Standardization guidelines stating that “weighting […] shall not be used in LCA studies intended to be used in comparative assertions intended to be disclosed to the public” [98]. Later the International Life Cycle Data System guidelines reported that “weighting may be necessary when trade-off situations occur in LCAs which are being used for comparing alternative products” [81]. The more recent United Nations Environment Programme Global Guidance mentions that “comparisons across impact categories should either […] or imply a value-based weighting step, which is recommended to be explicitly documented” [10, 99, 100]. The more recent international standards seem to lean towards the use of normalization and weighting, if the need is justified by increased interpretability [30].

This study acted on an opportunity to directly compare two pharmaceutical treatments, as these have both a measurable benefit and burden on the endpoint Human Health. The question is: do the health benefits outweigh the burdens? Additionally, normalization and weighting enabled the inclusion of the other two Areas of Protection in a common single score metric, thereby holistically capturing the benefits and burdens valued by policymakers and the general population.

Currently out of scope of the proposed framework is the treatment-related extinction of an entire pathogen species and its impact on Ecosystem quality. Further research is required on how the Human Health benefit would relate to the impact this extinction has on Ecosystem quality.

### 4.3 The proposed framework in relation to other studies

This study was not the first to quantify the Human Health benefit and the environmental footprint of a pharmaceutical treatment. Marsh et al. (2016) expands Health Technology Assessment (HTA) with environmental effects and assesses the treatment of type 2 diabetes, reporting patient outcomes in QALYs and the environmental impact in kg CO_2_ emissions [101–102]. While this study reports both the benefit of a pharmaceutical treatment and one particular environmental impact, both are not comparable through a common metric.

We identified several studies that did directly compare Human Health benefit to the Human Health burden attributable to the environment, albeit not on pharmaceutical treatments. Many such studies do not apply LCA but are focused on reducing air pollution through alternative (greener) means of transport. Apart from reducing emissions, there is a health benefit due to improved physical fitness of the population [103–108].

The studies that do apply LCA have a more varied range of subjects. Papa et al. (2013) discussed the Human Health benefits and burden, albeit reported in monetary terms, of the ozonation of WasteWater Treatment Plant (WWTP) effluents. Almeida et al. (2013) described the production of lead-free solder as opposed to solder that does contain lead. Gilbertson et al. (2014) compared the DALYs generated during the production of an H2S gas sensor to the potential DALYs saved upon implementation. Arvidsson et al. (2016) discussed positive and negative Human Health impacts in DALYs for three case studies: an airbag system, a catalytic converter of exhaust gases of cars and gold jewellery (DALYs from conflict minerals). Stylianou et al. (2016) assessed the environmental and nutritional Human Health effects in DALYs of adding one serving of milk to the average adult US diet. Afrinaldi et al. (2017) compared the environmental impacts of the diesel engine life cycle to the health benefit in the form of employee compensation, assuming workers use their wages to increase their quality of life.

Overall we can see a growing interest in recent years to include the benefit of products and services in Life Cycle Assessment.

### 4.4 Implications of the study

As demonstrated by the results of this study, it is meaningful to quantify the handprint of products and services in addition to the footprint as this shows not only the burdens associated with the life cycle but also considers possible benefits. These results provide hard data to answer the question: is the environmental burden of e.g. pharmaceutical treatments worth the value they create for society? One of the underlying issues of the RSBR is a question of Willingness to Accept: what is the footprint that we as society are willing to accept in order to enjoy the handprint? The RSBR may provide a more explicit answer to the question of many policymakers that currently have to compare environmental impacts on multiple midpoint categories with different units. The RSBR allows a direct comparison between the more interpretable Areas of Protection. Furthermore, it enables the handprint to be added to the same comparison.

Overall, this comprehensive approach may provide more information regarding the sustainability performance of products and services than classical LCA. In the case of the two evaluated pharmaceutical treatments, the single score performance was dominated by the handprint on the AoP Human Health. This suggests that future assessments of the environmental footprint in health care should also include the handprint when making a comparative assessment. Indeed, for any product or service that has a quantifiable handprint on one of the three AoPs, this should be taken into account in a holistic assessment.

### 4.5 Unanswered questions and future research

The methodology proposed in this study should be validated with case studies in other industrial sectors e.g. the handprint and footprint of nutritional diets or recycling [16, 109, 110]. The handprint for AoPs Ecosystems and Resources should also be included where possible. In most cases, the normalization of the handprint will be a challenge.

## Acknowledgments

The authors wish to thank Lorenzo Benini and Sue Ellen Taelman for their review of and valuable contributions to this study.

## Supporting information

S1 File Supporting information. (DOCX)
