# Correction: Quantifying the handprint—Footprint balance into a single score: The example of pharmaceuticals

**DOI:** 10.1371/journal.pone.0231596

**Published:** 2020-04-03

**Authors:** Sam Debaveye, Delphine De Smedt, Bert Heirman, Shane Kavanagh, Jo Dewulf

Drielsma et al. was incorrectly included as reference 83. As a result, all subsequent references are misnumbered. References 84–110 should be references 83–109.

The fifth sentence of the second paragraph in the 2.4.1 Part 1: Quantification of the footprint subsection of the Materials and Methods should have cited reference 39 instead of 83.

The correct sentence should read: Resources are expressed in US dollar ($), representing the surplus cost or marginal increase of the extraction cost for society of the next batch of resources, which increases as the more readily available resource deposits are depleted [39].
