# Supplementary material for: Quantifying the handprint—Footprint balance into a single score: The example of pharmaceuticals
Source: PLoS One. 2020 Feb 18;15(2):e0229235. doi: 10.1371/journal.pone.0229235 (PMC7028282; doi:10.1371/journal.pone.0229235)
Supplement: S1 File — (DOCX) [file pone.0229235.s001.docx]

**Supporting Information**

Quantifying the handprint – footprint balance into a single score: the example of pharmaceuticals

Sam Debaveye^1^, Delphine De Smedt^2^, Bert Heirman^3^, Shane Kavanagh^4^, Jo Dewulf^1^

*^1^ Research Group Environmental Organic Chemistry and Technology (EnVOC), Faculty of Bioscience Engineering, Ghent University, Campus Coupure, Ghent, Belgium*

*^2^ Department of Public Health, Ghent University, Campus UZ, Ghent, Belgium*

*^3^ Johnson & Johnson Environment, Health, Safety & Sustainability, Janssen Pharmaceutica NV, Beerse, Belgium*

*^4^ Health Economics, Janssen Pharmaceutica NV, Beerse, Belgium*

Corresponding author:

E-mail: samdebaveye@gmail.com (SD)

Number of pages: S8

Fig: S1-S3

Tables: S1-S5

# Environmental impact assessment of previous case studies

S1 Table: Environmental impact of paliperidone palmitate once-monthly treatment for 1000 patients in Belgium, expressed on three Areas of Protection.

|  | **Life Cycle Impact Assessment Results at endpoint** | | |
| --- | --- | --- | --- |
|  | **Human Health** | **Ecosystems** | **Resources** |
|  | **DALY** | **species.yr** | **$** |
| **Treatment: paliperidone palmitate 100 mg once-monthly** |  |  |  |
| Active Pharmaceutical Ingredient | 5.90E-03 | 2.71E-05 | 1.47E+02 |
| Drug Product | 2.41E-03 | 1.63E-05 | 7.88E+01 |
| Packaging | 3.02E-03 | 4.03E-05 | 1.27E+02 |
| Distribution & Supply | 4.87E-05 | 2.08E-07 | 1.35E+00 |
| End-of-Life disposal & drug fate | 3.50E-05 | -7.76E-07 | -6.37E+00 |
| GP visits | 4.36E-03 | 2.13E-05 | 1.43E+02 |
| Psychiatrist visits | 2.99E-01 | 1.46E-03 | 9.82E+03 |
| Ambulant care visits | 2.18E-03 | 1.07E-05 | 7.16E+01 |
| General hospital days | 2.45E-02 | 1.59E-04 | 7.98E+02 |
| Psychiatric hospital days | 1.71E-01 | 1.18E-03 | 8.16E+03 |
|  |  |  |  |
| **Treatment Interruption** |  |  |  |
| Active Pharmaceutical Ingredient | NA | NA | NA |
| Drug Product | NA | NA | NA |
| Packaging | NA | NA | NA |
| Distribution & Supply | NA | NA | NA |
| End-of-Life disposal & drug fate | NA | NA | NA |
| GP visits | 4.47E-02 | 1.85E-05 | 1.24E+02 |
| Psychiatrist visits | 2.89E-01 | 1.42E-03 | 9.51E+03 |
| Ambulant care visits | 5.75E-03 | 2.82E-05 | 1.89E+02 |
| General hospital days | 5.79E-02 | 3.75E-04 | 1.88E+03 |
| Psychiatric hospital days | 4.57E-01 | 3.16E-03 | 2.18E+04 |

Abbreviations: DALY, Disability-Adjusted Life Year; NA, not-applicable; yr, year

Negative impacts are associated with energy recovery after incineration.

S2 Table: Environmental impact of mebendazole donation for 8 million Vietnamese children for 5 years, expressed on three Areas of Protection.

|  | **Life Cycle Impact Assessment Results at endpoint** | | |
| --- | --- | --- | --- |
|  | **Human Health** | **Ecosystems** | **Resources** |
|  | **DALY** | **species.yr** | **$** |
| **Treatment: mebendazole 500 mg 6-monthly** |  |  |  |
| Active Pharmaceutical Ingredient | 4.97E+00 | 2.29E-02 | 2.02E+05 |
| Tablet formulation | 6.65E-01 | 4.11E-03 | 1.64E+04 |
| Packaging | 5.11E-02 | 2.74E-04 | 3.22E+03 |
| Distribution & Supply | 6.34E-02 | 2.23E-04 | 1.38E+03 |
| End-of-Life disposal & drug fate | 4.59E-03 | 1.22E-03 | 1.65E+01 |

Abbreviations: DALY, Disability-Adjusted Life Year; yr, year

# Normalization factors

S3 Table: Normalization Factors of ReCiPe Endpoint v1.11 [[1-3](#_ENREF_1)].

| Endpoint | Unit | World (2000) Individualist | World (2000) Hierarchist | World (2000) Egalitarian | PROSUITE (2010) |
| --- | --- | --- | --- | --- | --- |
| Human Health | DALY/yr | 9.20E+07 | 8.29E+07 | 1.47E+08 | 2.30E+08 |
| Ecosystems | species.yr/yr | 4.86E+06 | 5.58E+06 | 1.51E+07 | 5.08E+05 |
| Resources | $/yr | 5.99E+11 | 1.49E+12 | 1.49E+12 | 7.22E+11 |

Abbreviations: DALY, Disability-Adjusted Life Year; yr, year

The Normalization Factors from the PROSUITE project were expressed as DALY/yr/person, species.yr/yr/person and $/yr/person. Therefore we multiplied them with the number of people in the year 2010, obtained from the United Nations World Population Prospects [[4](#_ENREF_4)].

S4 Table: Normalization Factors of the Global Burden of Disease [[5](#_ENREF_5), [6](#_ENREF_6)].

| Region | Population | DALY |
| --- | --- | --- |
| World (2000) | 6.09E+09 | 2.80E+09 |
| World (2005) | 6.49E+09 | 2.75E+09 |
| World (2010) | 6.89E+09 | 2.67E+09 |
| World (2015) | 7.31E+09 | 2.66E+09 |

Abbreviations: DALY, Disability-Adjusted Life Year

# Weighting factors

Weighting Factors (WF) were obtained from Eco-Indicator 99, [Itsubo et al. (2015)](#_ENREF_7) and the European Commission (EC) Joint Research Centre (JRC) report on Weighting Factors for the Environmental Footprint [[8](#_ENREF_8)]. For the first two WF were available at endpoint directly. For the EC JRC report weights were only provided at midpoint, while at endpoint the categories were compared to each other on importance using scores on 100. As we aimed to include WF from different sources, we converted the midpoint weights to endpoint, visible in S1 Fig. The scores on 100 were normalized to weights, displayed in S2 Fig. Both approaches should be seen as a way to include more sensitivity in the results.


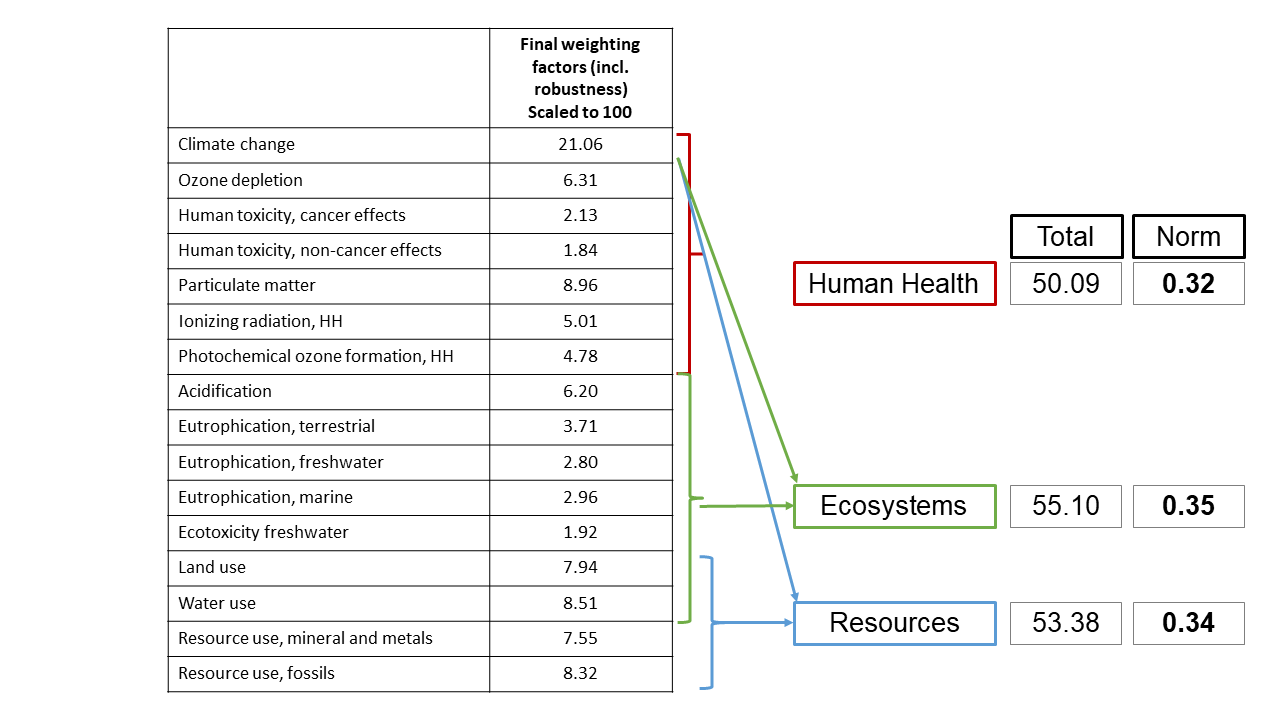


S1 Fig: The midpoint weights were aggregated to endpoint by summing up the weighting factors that contribute to each endpoint, after which the sums were normalised to 1. Fig adapted from [Sala et al. (2018)](#_ENREF_9)


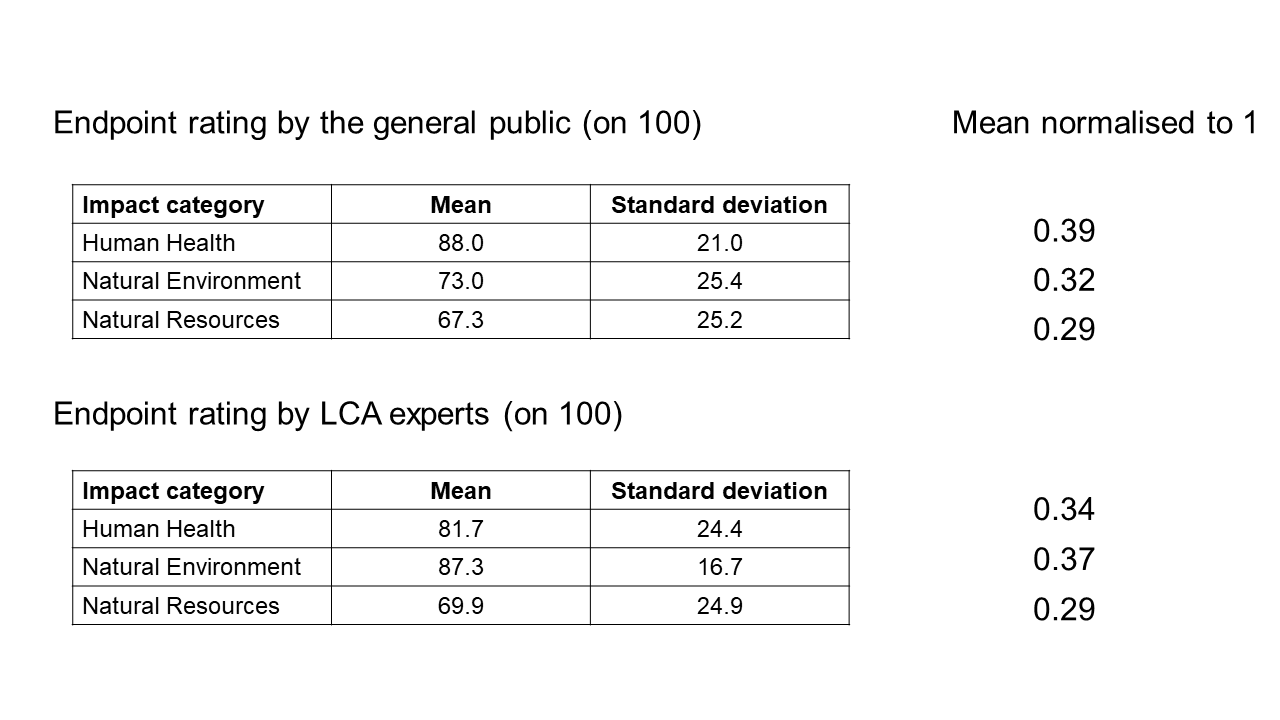


S2 Fig: The rating of endpoints by the general public and LCA experts. Scores on 100 were normalised to 1. Fig adapted from [Sala et al. (2018)](#_ENREF_9)

S3 Fig: Weighting sets applied in this study.

S5 Table: Main weighting sets and subsets applied [[8](#_ENREF_8), [7](#_ENREF_7), [9](#_ENREF_9)].

| Eco-indicator 99 | | Itsubo et al., 2015 | | EC JRC Environmental Footprint | |
| --- | --- | --- | --- | --- | --- |
| Subset | Weight | Subset | Weight | Subset | Weight |
| Default | Human Health: 0.40  Ecosystems: 0.40  Resources: 0.20 | GBR | Human Health: 0.40  Ecosystems: 0.40  Resources: 0.20 | Midpoint to endpoint | Human Health: 0.32  Ecosystems: 0.35  Resources: 0.34 |
| Individualist | Human Health: 0.55  Ecosystems: 0.25  Resources: 0.20 | FR | Human Health: 0.39  Ecosystems: 0.43  Resources: 0.18 | Importance general public | Human Health: 0.39  Ecosystems: 0.32  Resources: 0.29 |
| Hierarchist | Human Health: 0.30  Ecosystems: 0.40  Resources: 0.30 | GER | Human Health: 0.26  Ecosystems: 0.54  Resources: 0.20 | Importance LCA experts | Human Health: 0.34  Ecosystems: 0.37  Resources: 0.29 |
| Egalitarian | Human Health: 0.30  Ecosystems: 0.50  Resources: 0.20 | ITA | Human Health: 0.46  Ecosystems: 0.40  Resources: 0.14 |  |  |
|  |  | USA | Human Health: 0.52  Ecosystems: 0.40  Resources: 0.08 |  |  |
|  |  | CAN | Human Health: 0.43  Ecosystems: 0.36  Resources: 0.21 |  |  |
|  |  | AUS | Human Health: 0.34  Ecosystems: 0.49  Resources: 0.17 |  |  |
|  |  | JPN | Human Health: 0.30  Ecosystems: 0.47  Resources: 0.23 |  |  |
|  |  | KOR | Human Health: 0.45  Ecosystems: 0.31  Resources: 0.24 |  |  |
|  |  | ARG | Human Health: 0.51  Ecosystems: 0.15  Resources: 0.34 |  |  |
|  |  | SAU | Human Health: 0.61  Ecosystems: 0.23  Resources: 0.16 |  |  |
|  |  | RUS | Human Health: 0.38  Ecosystems: 0.37  Resources: 0.24 |  |  |
|  |  | MEX | Human Health: 0.42  Ecosystems: 0.28  Resources: 0.30 |  |  |
|  |  | TUR | Human Health: 0.41  Ecosystems: 0.30  Resources:0.28 |  |  |
|  |  | BRA | Human Health: 0.57  Ecosystems: 0.23  Resources: 0.20 |  |  |
|  |  | ZAF | Human Health: 0.58  Ecosystems: 0.21  Resources: 0.20 |  |  |
|  |  | CHN | Human Health: 0.40  Ecosystems: 0.34  Resources: 0.26 |  |  |
|  |  | IDN | Human Health: 0.61  Ecosystems: 0.17  Resources: 0.22 |  |  |
|  |  | IND | Human Health: 0.49  Ecosystems: 0.26  Resources: 0.26 |  |  |

# Bibliography

1. Goedkoop M, Heijungs R, Huijbregts MAJ, De Schryver A, Struijs J, van Zelm R. ReCiPe 2008: A life cycle impact assessment method which comprises harmonised category indicators at the midpoint and the endpoint level. Report I: Characterisation. Dutch ministry of Housing, Spatial Planning and the Environment, 2009.

2. Gaasbeek A, Meijer E. PROSUITE Handbook on a novel methodology for the sustainability impact assessment of new technologies. 2013.

3. Laurent A, Hauschild MZ, Golsteijn L, Simas M, Fontes J, Wood R. Deliverable 5.2: Normalisation factors for environmental, economic and socio-economic indicators. Copenhagen: 2013.

4. United Nations. World Population Prospects 2017 2018. Available from: <https://esa.un.org/unpd/wpp/Download/Standard/Population/>.

5. Vos T. Global, regional, and national incidence, prevalence, and years lived with disability for 310 diseases and injuries, 1990–2015: a systematic analysis for the Global Burden of Disease Study 2015. Lancet. 2016;388(10053):1545-602. doi: <https://doi.org/10.1016/S0140-6736(16)31678-6>.

6. WHO. WHO methods and data sources for global burden of disease estimates 2000-2015. Geneva: World Health Organization, 2017.

7. Itsubo N, Murakami K, Kuriyama K, Yoshida K, Tokimatsu K, Inaba A. Development of weighting factors for G20 countries—explore the difference in environmental awareness between developed and emerging countries. Int J Life Cycle Assess. 2015:1-16. doi: 10.1007/s11367-015-0881-z.

8. Goedkoop M, Spriensma R. The Eco-indicator 99 A damage oriented method for Life Cycle Impact Assessment - Methodology Report. Amersfoort: PRé Consultants, 1999.

9. Sala S, Cerutti AK, Pant R. Development of a weighting approach for the Environmental Footprint. Luxembourg: European Commission, 2018.
